# Unleashing the potential of Health Promotion in primary care—a scoping literature review

**DOI:** 10.1093/heapro/daae044

**Published:** 2024-05-25

**Authors:** Adela Bisak, Martin Stafström

**Affiliations:** Faculty of Medicine, Lund University, Jan Waldenströms gata 35, 214 28 Malmö, Sweden; Division of Social Medicine and Global Health, Lund University, Jan Waldenströms gata 35, 214 28 Malmö, Sweden

**Keywords:** salutogenesis, scoping literature review, primary care, healthy individuals

## Abstract

The purpose of this study is to gain a better understanding of the role and extent of health promotion lifestyle interventions targeting adults in primary care, and especially those who are considered overall healthy, i.e. to study the outcomes of research applying salutogenesis. We performed a literature review, with three specific aims. First, to identify studies that have targeted the healthy population in intervention within the primary health care field with health promotion activities. Second, to describe these interventions in terms of which health problems they have targeted and what the interventions have entailed. Third, to assess what these programs have resulted in, in terms of health outcomes. This scoping review of 42 studies, that applied salutogenesis in primary care interventions shows that health promotion targeting healthy individuals is relevant and effective. The PRISMA-ScR guidelines for reporting on scoping review were used. Most interventions were successful in reducing disease-related risks including CVD, CVD mortality, all-cause mortality, but even more importantly success in behavioural change, sustained at follow-up. Additionally, this review shows that health promotion lifestyle interventions can improve mental health, even when having different aims.

**Contribution to Health Promotion**

- This article describes the importance of including healthy individuals in health promotion activities, applying salutogenesis, as there are significant positive health outcomes effects if they participate in health interventions.
- The study amplifies that the prevention paradox should always be considered when designing health promotion interventions.
- This article shows that the greatest effects when targeting healthy individuals are found in lower all-cause mortality and CVD risks, mainly because these programs manage to lead to long-lasting lifestyle changes.

## INTRODUCTION

Health Promotion is, according to Nutbeam and Muscat (2021, p. 1580), ‘[…] the process of enabling people to increase control over, and to improve their health’. This process entails a comprehensive approach to change on all levels, from structures to individuals, improving health mainly through addressing the social determinants of health.

Whereas the most overarching processes are initiated on a structural level through global and national health policies (Cross *et al*., 2020), health promotion strategies are also widely employed in health interventions targeting individuals. It could entail smoking cessation programs, weight loss programs and adolescent alcohol use, just to mention some common health outcome target areas (Green *et al.*, 2019). Even when deploying health promotion strategies at a national policy level, it is not uncommon that the programs designed to target individuals and groups are more inspired by pathogenesis, rather than salutogenesis (Nutbeam and Muscat, 2021).

A widespread strategy in the latter programs is that individuals are screened for a need to receive an intervention, so-called secondary or indicated prevention programs, where those who report a riskier lifestyle, or test worse on psychometric or biometric indicators are eligible for receiving the intervention, and those not having the same risks are excluded from the program on the premise that they are, based on study protocol definitions, healthy individuals.

Based on the principles of salutogenesis, this is a somewhat inappropriate approach. Within the strategy of health promotion, it is assumed that all people, no matter their level of risk, would find feedback on their health valuable. Those in need of change should receive the necessary resources and tools to change, whereas those who do not have to change should have their lifestyles positively reinforced. In addition, the prevention paradox (Rose, 1981) postulates that it is important to address the majority, as there will be plenty of adverse health outcomes stemming from them. In conventional indicated prevention programs, attention to those who are non-eligible for interventions is, thus, often completely disregarded.

One common arena for such programs is primary health care. There is a wide range of evidence-based programs that have shown efficacy in reducing the health risks among those who have the riskiest lifestyles in relation to, e.g. alcohol use (Beyer *et al*., 2019), smoking (Cantera *et al*., 2015), depression (Bortolotti *et al*., 2008), diabetes (Galaviz *et al*., 2018) and cardiovascular diseases (Álvarez-Bueno *et al.*, 2015). The at-risk groups vary across the different diseases, but a vast majority of patients targeted in the above studies were identified after screening as non-eligible to participate in the intervention in question. From this follows that a large number of individuals do not receive any substantial health information, nor are their health outcomes measured as they are not included in the intervention. From a health promotion perspective, this seems like a lost opportunity. Additionally, this raises the question of whether a healthy population is systematically disadvantaged compared to those individuals at high risk, which might point to some less-known health inequities or disparities (Braveman, 2006) present in primary care.

In order to gain a better understanding of the effects of health promotion as an overall approach, and to understand the implications of the prevention paradox, it would be pertinent to include the non-eligible group in both the feedback loop—mainly offering them structured positive reinforcement—and to subsequently measure their health and attributed lifestyles.

The purpose of this study is to gain a better understanding of the role and extent of health promotion lifestyle interventions targeting adults in primary care, especially those who are considered overall healthy. More precisely we aim to assess to what extent health promotion practices in primary care address healthy individuals, not only those who need to undergo a lifestyle change. In order to do so, we performed a literature review, with three specific aims. First, to identify studies that have only targeted the healthy population, or healthy population in addition to high-risk group in intervention within the primary health care field with health promotion activities. Second, to describe these interventions in terms of which health problems they have targeted and what the interventions have entailed. Third, to assess what the initiatives published in the research literature have resulted in terms of health outcomes.

## METHODS

Due to the width of the topic and study designs we chose to perform a scoping review, with the aim of summarizing and disseminating previous research and identifying research gaps in the literature (Arksey and O’Malley, 2005). The search process was iterative and non-linear, reflecting upon the results from the literature search at each stage and then repeating steps where necessary to cover the literature more comprehensively (Arksey and O’Malley, 2005).

A few terms demand some further definition within the scope of this review*. Healthy individual* is a fluid term varying across different studies and contexts, yet it is a key concept in this particular study. The term involves those without chronic disease, who are indicated as not being of an elevated risk of developing a disease linked to the health outcome they have been screened for, but they could very well be at risk for diseases beyond the scope of the study they have been examined within. *Primary health care* may in this review indicates different types of settings from the most common one relating to general practitioners and family doctors to occupational medicine or periodical work-related health check-ups but also dental health care. *Health promotion interventions* in this study are understood as interventions that aim to keep people healthy longer, by providing positive feedback in relation to current and new health behaviours, rather than controlling health status by medication use.

### Search strategy

The search was done across two databases PubMed and Embase, by combining different strings related to keywords ‘health promotion’ and ‘primary care’, while the rest of the strings varied, more specific search queries are available in Supplementary Appendix A. The search was conducted during June and July 2023 and consisted of publications dated between July 2008 and July 2023 (i.e. the last 15 years). Additional studies were identified manually from references of the included articles and by ‘See all similar articles’ option in PubMed and ‘similar records’ in Embase. The article titles were scanned from databases, followed by screening titles and abstracts through the Covidence software, and then finally the full articles were read. Results were filtered for adult humans, defined as age 18–75, abstracts being available and the studies were authored in English.

Articles were included if (i) the population consisted of working-age adults, (ii) the population included those screened as healthy within a whole sample followed by an intervention or interventions ideally at follow-up, (iii) the study focused on primary prevention (iv) the study focused on lifestyle interventions, (v) the study examined lifestyle-related behaviours. Exclusion criteria for papers were (i) focused on children—below the age of 18 or elderly, (ii) addiction behaviours, (iii) excluding healthy individuals from intervention after screening or using them exclusively in the control group, (iv) using only high-risk population as healthy, (v) promoting only mental health, (vi) secondary prevention, (vii) screening is the only intervention, (viii) reviews and study protocols.

After full-text screening, the data charting process for reviewing, sorting and documenting information (Arksey and O’Malley, 2005) was done using Covidence, Data Extraction version 2 recommended for scoping reviews. The Data Extraction Template included columns for article title, author, country in which the study was conducted, methods (aim, design, population description, inclusion and exclusion criteria) intervention description, outcome measures, relevant results, follow-up (yes/no), study setting (primary care, worksite/occupational, population-based), study category (lifestyle, physical activity and diet, cardiovascular disease, alcohol consumption) and a field for additional notes where needed.

Due to great inconsistencies between studies in the design, populations and outcomes, critical appraisal of individual sources of evidence—an optional step in PRISMA-ScR (Tricco *et al*., 2018) guideline list was not done, although concerning research aim it would be useful for assessing the quality of evidence. Although exclusion/inclusion criteria were respected, what was considered as ‘healthy’, ‘middle-’ or ‘high-risk population’ differed significantly in studies, due to differences in definition of terms. Moreover, this decision was made as the AMSTAR tool would not be an adequate choice due to the inclusion of a non-randomized design, and although the AMSTAR 2 tool could potentially be used, this review also included several economic evaluations and follow-ups (Supplementary Table S1 for more details), or indicators differing highly across studies.

For the synthesis of results (Tricco *et al*., 2018), the studies were grouped by the type of the outcome—disease, i.e. CVD or lifestyle/behaviour: physical activity and diet or alcohol consumption. Furthermore, the studies were summarized by setting, risk group and follow-up. None of the systematic reviews with similar research aims were detected during the search.

## RESULTS

**Fig. 1:**
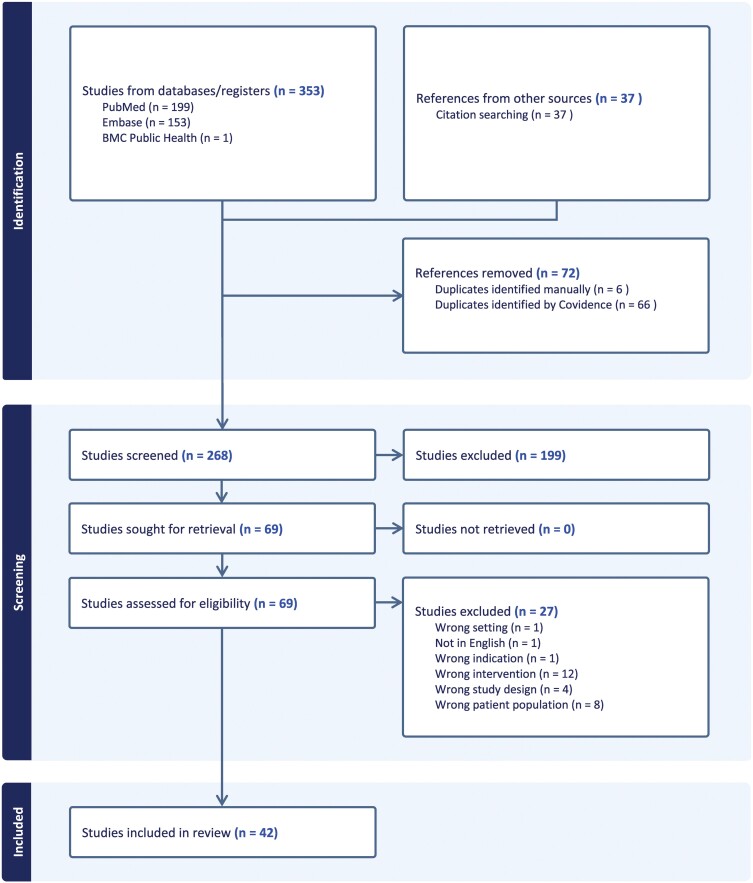
PRISMA of full screening strategy.

The selection of sources of evidence (Tricco *et al*., 2018) was done as described: 353 references were imported for screening, 72 duplicates were removed, 268 studies were screened against title and abstract during which 198 studies were excluded while 69 studies were assessed for full-text eligibility, when 27 studies were excluded: 12 for wrong intervention, 8 for wrong patient population, 4 for wrong study design 1 was not in English, 1 for wrong indication and 1 for wrong setting, after which 42 studies were included. PRISMA of full screening is found in Figure 1.

### Lifestyle interventions

A summary of the study setting, samples and the main outcomes of the 42 studies analysed in this scoping review is presented in Supplementary Table S1.

In general, the intervention studies analysed here had different main strategies, including: individually tailored programs (Doumas and Hannah, 2008; Gram *et al*., 2012; Watson *et al*., 2015) risk-based, group-based (Recio-Rodriguez *et al*., 2016) or mixed variants (Matano *et al*., 2007; Matzer *et al*., 2018).

#### Cardiovascular health

We found several different lifestyle interventions targeting CVD risk. There were a set of programs that addressed physical activity in the workplace, which significantly reduced the CVD risk in healthy participants adhering to the program (Gram *et al*., 2012; Dalager *et al*., 2016; Eng *et al*., 2016; Biffi *et al*., 2018). In primary care, an observational study by Journath *et al*. (2020), showed an association between healthy participant participation in a CVD prevention programme promoting physical activity and a healthy lifestyle with lower risk of CV events (12%), CV mortality (21%) and all-cause mortality (17%) after 20 years of follow-up.

Similarly, we found interventions in primary care settings that led to changes in physical activity and dietary patterns among all participants—not only those at high risk of CVD morbidity and mortality. These studies described generally decreased CVD risks (Richardson *et al*., 2008; Buckland *et al*., 2009; Nguyen *et al*., 2012; Gibson *et al*., 2014; Bo *et al*., 2016; Lidin *et al*., 2018; Lingfors and Persson, 2019), CVD-related mortality (Blomstedt *et al*., 2011; Persson *et al*., 2015; Jeong *et al*., 2019) and all-cause mortality (Blomstedt *et al*., 2015; Bo *et al*., 2016; Bonaccio *et al*., 2019).

In a prospective observational study on healthy individuals and those with CVD conducted by Lidin *et al*. (2018), the prevalence within the sample at risk of CVD decreased significantly at 12-month follow-up by 15%. In several studies, the changes in health behaviours among the participants showed to be sustained in follow-ups conducted after intervention discontinuation (Buckland *et al*., 2009; Gibson *et al*., 2014; Baumann *et al*., 2015; Blomstedt *et al*., 2015; Lidin *et al*., 2018), while some cardiovascular risk factors, such as salty diets and smoking, showed evidence of significant decrease in a relatively short period (Nguyen *et al*., 2012).

#### Physical activity and diet

In interventions addressing physical activity and diet, it was evident that healthy individuals were more likely to adhere to physical activity interventions (Dalager *et al*., 2016; Biffi *et al*., 2018; Jeong *et al*., 2019) compared to those with a disease. One community-based walking intervention (Yang and Kim, 2022) affected not only the level of physical activity significantly but also a positive overall change towards a health-promoting lifestyle and decreased perceived stress. Similarly, several mental health measures including general mental health (Oude Hengel *et al*., 2014), anxiety and depression (Gibson *et al*., 2014) and stress (Lingfors *et al*., 2009; Matzer *et al*., 2018) in participants improved during interventions and at follow-up when targeting physical activity and diet.

Additionally, concerning physical activity and diet outcomes, there were a higher feasibility of uptake among participants in health promotion programs compared to those only receiving standard care in primary care (Lingfors *et al*., 2009; Zabaleta-Del-Olmo *et al*., 2021). Anokye *et al.* (2014) argued that brief advice intervention was more effective—leading to 466 QALYs gained, compared to standard care—implying greater cost-effectiveness.

Healthier lifestyles were also maintained at the follow-up. Reduction in risk factors was found to be sustained in follow-ups at 12 months (Gibson *et al*., 2014) or improvements in dietary outcomes over 5 years (Baumann *et al*., 2015), and sustained lower blood pressure over 6 years (Eng *et al*., 2016).

Several interventions promoting physical activity in primary care settings showed significant results in increasing it in all patients, not only in those with chronic disease diagnosis (Robroek *et al*., 2010; Gram *et al*., 2012; Hardcastle *et al*., 2012; Viester *et al*., 2015; Byrne *et al*., 2016; Dalager *et al*., 2016; Eng *et al*., 2016; Recio-Rodriguez *et al*., 2016; Biffi *et al*., 2018; Matzer *et al*., 2018; Yang and Kim, 2022), and similar patterns were also found concerning a change towards a healthier diet (Lingfors *et al*., 2009; Wendel-Vos *et al*., 2009; Robroek *et al*., 2010; Baumann *et al*., 2015; Viester *et al*., 2015; Bo *et al*., 2016; Byrne *et al*., 2016; Kosendiak *et al*., 2021).

There was disagreement among the above studies in relation to the effectiveness of these interventions among healthy individuals. For example, in the case of implementing a Mediterranean diet, one report argued that a healthy diet should be prioritized, indicating significant hazard ratios (HR) of attaining a Mediterranean diet for all-cause mortality (HR = 0.83), CV mortality (HR = 0.75) and CV events (HR = 0.79) among low-risk individuals (Bo *et al*., 2016). Others, however, claimed that there was no evidence of healthier participants being more susceptible to changes in physical activity and diet (Robroek *et al*., 2010).

#### Alcohol consumption

Interventions aimed at decreasing alcohol consumption were divided between those being most effective in high-risk drinkers (Doumas and Hannah, 2008; Kirkman *et al*., 2018), and both moderate and low-risk drinkers (Matano *et al*., 2007). These interventions were, at large, seen as cost-saving (Watson *et al*., 2015) and feasible in primary care (Neuner-Jehle *et al*., 2013). Some studies found a sustained decrease in alcohol consumption in those adhering to the interventions, compared to the control groups at 1 (Pemberton *et al*., 2011) and 4 months after the intervention (Kirkman *et al*., 2018), whereas others failed to find a significant difference between groups.

### Intervention setting

The interventions took place in primary care settings, though these were either in community-based or occupational settings. The findings suggested that there were some discrepancies between these different settings.

When it comes to a community-based setting, the difference is made between interventions conducted on a sample of those visiting primary health care or a sample representative for a population of one community—town, or region. Primary care community-based studies tended to either include participants who were primary care visitors with a long follow-up period, or interventions conducted in primary care clinic centres with a shorter follow-up period, most often using experimental design, sampling individuals living in the community that did not necessarily had an intention to seek care (Richardson *et al*., 2008; Hardcastle *et al*., 2012; Nguyen *et al*., 2012; Grunfeld *et al*., 2013; Baumann *et al*., 2015; Bo *et al*., 2016; Lidin *et al*., 2018; Zabaleta-Del-Olmo *et al*., 2021).

Overall, the community-based studies were conducted on a sample representative for a population of a smaller community (Kosendiak *et al*., 2021; Yang and Kim, 2022), region (Lingfors *et al*., 2009; Wendel-Vos *et al*., 2009; Gibson *et al*., 2014; Persson *et al*., 2015; Bonaccio *et al*., 2019; Jeong *et al*., 2019; Lingfors and Persson, 2019; Journath *et al*., 2020) or a country (Buckland *et al*., 2009; Blomstedt *et al*., 2011; Neuner-Jehle *et al*., 2013), often followed by a longer follow-up period. Finally, some studies were evaluations of previous interventions (Richardson *et al*., 2008; Anokye *et al*., 2014).

Worksite interventions comprised of different occupational roles, often including several of those in the same sample (Eng *et al*., 2016), or segmenting based on how physically active the occupation was, e.g. office workers (Dalager *et al*., 2016), construction workers (Gram *et al*., 2012; Oude Hengel *et al*., 2014; Viester *et al*., 2015), sailors (Hjarnoe and Leppin, 2013), farmers (van Doorn *et al*., 2019) or simply more active individuals (Biffi *et al*., 2018). This had the implication that approaches to intervention differed widely across the studies.

Several interventions were conducted online using a web-based interface, while others were in a professional setting (Matano *et al*., 2007; Doumas and Hannah, 2008; Robroek *et al*., 2010; Pemberton *et al*., 2011; Khadjesari *et al*., 2014) or in some cases community-based (Recio-Rodriguez *et al*., 2016; Kirkman *et al*., 2018).

### Categorization of risk among participants

Many studies applied specific risk criteria based on the participants’ morbidity risks: including groups of low, middle, high risk (Persson *et al*., 2015; Bo *et al*., 2016; Lingfors and Persson, 2019), low and high risk (Baumann *et al*., 2015), middle and high risk (Gibson *et al*., 2014). While some did not distinguish between risk groups (Wendel-Vos *et al*., 2009; Blomstedt *et al*., 2011; Byrne *et al*., 2016; Journath *et al*., 2020). In some studies, however, the protocol included mixed populations of those who were healthy and those who had a chronic disease (Anokye *et al*., 2014; Bonaccio *et al*., 2019). Finally, different studies came up with their own meaning of ‘healthy individual’ or ‘healthy population’ based on the health problem they addressed, i.e. having a sedentary lifestyle or high alcohol consumption. Other criteria for being a part of a healthy population were having a high risk for a disease, one or several risks but not the disease itself, or being above a reference value without having a diagnosis.

### Ethical implications of healthy controls

Some interventions were screening-result-based, meaning that there was a difference in the treatment of those with good health and those with some complications. In other words, although not excluding healthy individuals, the study protocol included healthy individuals partially receiving full treatment, in the intervention. Studies that excluded those who were healthy from the sample after screening or used them as a control group were excluded from this review. However, some included studies had a healthy control group. Overall, the studies included in this review did not discuss the ethical implications of including healthy populations as controls, or when that was the case, the ethical impact of excluding healthy participants from an intervention.

## DISCUSSION

This scoping review speaks not only of the role and extent of health promotion for healthy individuals in primary care but also of the importance and effects it has on population health. The results showing the association of lifestyle interventions with CVD risk show great implications for future use in primary care, different contexts and feasibility. Physical activity interventions were additionally found to be related to some improvements in mental health.

Interventions aimed at alcohol consumption were found successful in decreasing the amount of drinking sustainably, while the main discussion was based on whether they should be aimed at high-risk only, or at middle- and low-risk drinkers as well, due to mixed results in said groups. The majority of interventions were based in a worksite setting, meaning that this context might be useful for tackling the issue. This approach showed that outcomes might be beneficial even when not reaching the primary goal. Examples of this are findings showing that although not reducing CVD risk, changes in health behaviours were sustained in follow-up (Baumann *et al*., 2015), less drastic changes decreasing CVD risk in the healthy population (Buckland *et al*., 2009) and beneficial effects of physical activity intervention on worker’s health without an overall increase in physical activity (McEachan *et al*., 2011). Finally, in most cases, as mentioned, changes in health behaviours were associated with changes in CVD risk.

Some interventions showed that health promotion benefits could be even bigger (Bo *et al*., 2016) or that adherence is higher in healthy participants (Dalager *et al*., 2016; Biffi *et al*., 2018; Jeong *et al*., 2019), while other authors disagree (Robroek *et al*., 2010). This could be traced to the topic of prioritising primary care for healthy, versus only those at high risk/ already with a disease—secondary care approach according to this review definitions. Designing interventions only for high-risk can make them less successful in healthy participants, as displayed in a study by Blomstedt *et al*. (2011) where self-rated health decreased in 21% of the good baseline health participants at the 10-year follow-up. Furthermore, from the Rose’s (1981) term of prevention paradox—a great benefit for the population can be almost non-existent for an individual, while if we only focus on high-risk cases, many individuals at low-risk can mean worse health outcomes compared to a small number at high-risk (Rose, 2001). In other words, by focusing only on high-risk population, the downsides are care that can be less efficient, less feasible, more expensive and lead to worse health outcomes. This choice should not be exclusive, as excluding either populations can cause ethical concerns. However, this article gives priority to early prevention, by health promotion for healthy individuals in primary care. Additionally, if it is shown that ‘*Systems based on primary care have better population health, health equity, and health care quality, and lower health care expenditure…*’ (Stange *et al.*, 2023), different treatment of those who are currently healthy presents an obstacle worth mentioning for achieving health equity in primary care. Furthermore, the role of promoting health to healthy populations and their inclusion in interventions is crucial for improving population health in the future.

Articles focusing on smoking cessation, alcoholism, substance misuse interventions were excluded from this scoping review as they represent addictions and are therefore different from lifestyle interventions. Originally, oral health and dental care interventions were to be included, but there were not enough studies matching the scoping review inclusion requirements.

As expected, the process of finding articles appropriate for inclusion was challenging. Even when the inclusion criteria, at first glance, were satisfied, most studies we came across had excluded healthy participants from the sample after screening for being asymptomatic or not having enough risk factors. They were, however, often a part of a control group, and usually received standard care or no care at all. This approach puts healthy individuals in a vulnerable position, by not addressing their needs to change lifestyles that eventually could contribute to an early death or becoming unwell. Our findings suggest that interventions that include healthy individuals could improve quality of life and health status both at the population and individual levels.

Due to studies using different risk criteria, as well as including many study designs and topics, it was hard to make general conclusions. Nevertheless, as a scoping review, we mapped the area of research by identifying the gaps in the evidence base, and summarizing and disseminating research findings (Arksey and O’Malley, 2005), instead of appraising the quality of evidence in different studies.

Concerning the above, a big research gap was detected in studies focusing on, or even including healthy populations. Furthermore, there is a lack of a coherent or comprehensive methodology in assessing the effects of what is considered health promotion, which calls for a more specific approach and a clear definition of the term. Additionally, the question of intervention staff skills should be raised. Is it necessary that health promotion interventions should be conducted by clinically trained professionals or, innovatively, by staff trained in the topic at hand when possible? Another aspect that is important to problematize is whether it is ethical to exclude healthy individuals in health promotion intervention studies even if they would benefit from participating if included? Furthermore, if healthy individuals are systematically discriminated (Braveman, 2006), receive worse treatment and have the risk of worse health outcomes in the future, it is critical to include them in interventions for achieving better health of populations. This has great practical implications for primary care. Similarly, from a cost-benefit perspective, research should address if excluding healthy individuals might affect the cost-effectiveness of health promotion interventions.

An apparent limitation within this review is the culturally uniform sample of studies. Most studies that we were able to identify were a result of research in the global north, with a strong emphasis on either North America or the EU. Only two studies were from less affluent settings in Southeast Asia (Nguyen *et al*., 2012; Bo *et al*., 2016). Given that the findings suggest that these interventions are cost-effective and do not require substantial investments, these programs could have great potential in low-resource settings if more systematically researched.

## CONCLUSION

This scoping review of 42 studies applying salutogenesis in primary care interventions shows that health promotion targeting healthy individuals is relevant and effective. Most interventions were successful in reducing disease-related risks including CVD, CVD mortality, all-cause mortality, but even more importantly success in behavioural change, sustained at follow-up. Additionally, this review shows that health promotion lifestyle interventions can improve mental health, even when having different aims.

## SUPPLEMENTARY MATERIAL

Supplementary material is available at *Health Promotion International* online.

daae044_suppl_Supplementary_Appendix

daae044_suppl_Supplementary_Table_S1

## Data Availability

The data underlying this article are available in the article and in its online supplementary material.
