# Supplementary material for: Unleashing the potential of Health Promotion in primary care—a scoping literature review
Source: Health Promot Int. 2024 May 25;39(3):daae044. doi: 10.1093/heapro/daae044 (PMC11127486; doi:10.1093/heapro/daae044)
Supplement: daae044_suppl_Supplementary_Appendix [file daae044_suppl_supplementary_appendix.docx]

**Appendix A**

**Full search strategy and queries from PubMed and Embase**

**PubMed**

1. (((primary health care[MeSH Terms]) OR ("primary health care"[Title/Abstract])) OR ("primary care"[Title/Abstract])) AND ((health promotion[MeSH Terms]) OR ("health behavior"[MeSH Terms])) OR (healthy people programs[MeSH Terms]) OR ("health promotion"[Title/Abstract] OR "health behavior"[Title/Abstract] OR "healthy people programs"[Title/Abstract]))

2. ((("primary health care"[MeSH Terms] OR "primary health care"[Title/Abstract] OR "primary care"[Title/Abstract]) AND (health promotion[MeSH Terms] OR ("health promotion"[Title/Abstract] AND ("prevention"[Title/Abstract])) OR (prevention[MeSH Terms])) AND (cardiovascular disease[MeSH Terms])) OR ("cardiovascular disease"[Title/Abstract]) AND ((adult[Filter] OR middleaged[Filter]) AND (2007:2023[pdat])))

3. ((("primary health care"[MeSH Terms] OR "primary health care"[Title/Abstract] OR "primary care"[Title/Abstract]) AND (health promotion[MeSH Terms] OR ("health promotion"[Title/Abstract] AND ("prevention"[Title/Abstract])) OR (prevention[MeSH Terms])) AND (intervention [MeSH Terms])) OR program [MeSH terms] OR ("intervention"[Title/Abstract]) OR "program"[Title/Abstract] AND ((adult[Filter] OR middleaged[Filter]) AND (2007:2023[pdat])))

4. ((("primary health care"[MeSH Terms] OR "primary health care"[Title/Abstract] OR "primary care"[Title/Abstract]) AND (health promotion[MeSH Terms] OR ("health promotion"[Title/Abstract] AND ("prevention"[Title/Abstract])) AND (occupational health [MeSH Terms])) OR (worksite [MeSH Terms])) OR ("occupational health "[Title/Abstract]) OR "worksite"[Title/Abstract] AND ((adult[Filter] OR middleaged[Filter]) AND (2007:2023[pdat])))

5. ((("primary health care"[MeSH Terms] OR "primary health care"[Title/Abstract] OR "primary care"[Title/Abstract]) AND (health promotion[MeSH Terms] OR ("health promotion"[Title/Abstract] AND ("prevention"[Title/Abstract])) AND (occupational health [MeSH Terms])) OR ("occupational health "[Title/Abstract]) OR "check-up"[Title/Abstract] AND ((adult[Filter] OR middleaged[Filter]) AND (2007:2023[pdat])))

6. ((("primary health care"[MeSH Terms] OR "primary health care"[Title/Abstract] OR "primary care"[Title/Abstract]) AND (health promotion[MeSH Terms] OR ("health promotion"[Title/Abstract] AND ("prevention"[Title/Abstract])) AND (occupational health [MeSH Terms])) OR (worksite [MeSH Terms])) AND (alcohol [MeSH Terms]) OR (alcohol consumption [MeSH Terms]) OR ("occupational health "[Title/Abstract]) OR "worksite"[Title/Abstract] AND "alcohol" [Title/Abstract] AND ((adult[Filter] OR middleaged[Filter]) AND (2007:2023[pdat])))

7. ((("primary health care"[MeSH Terms] OR "primary health care"[Title/Abstract] OR "primary care"[Title/Abstract]) AND (health promotion[MeSH Terms] OR ("health promotion"[Title/Abstract] AND ("prevention"[Title/Abstract])) OR (prevention[MeSH Terms])) AND (dental [MeSH Terms])) OR (oral health [MeSH Terms]) OR ("dental"[Title/Abstract]) OR ("oral health"[Title/Abstract]) AND ((adult[Filter] OR middleaged[Filter]) AND (2007:2023[pdat])))

8. ((("primary health care"[MeSH Terms] OR "primary health care"[Title/Abstract] OR "primary care"[Title/Abstract]) AND (health promotion[MeSH Terms] OR ("health promotion"[Title/Abstract] AND ("prevention"[Title/Abstract])) OR (prevention[MeSH Terms])) AND (health behavior[MeSH Terms])) OR ("health behavior"[Title/Abstract]) AND ((adult[Filter] OR middleaged[Filter]) AND (2007:2023[pdat])))

9. (((((("primary health care"[MeSH Terms] OR "primary health care"[Title/Abstract] OR "primary care"[Title/Abstract]) AND ("health promotion"[MeSH Terms] OR ("health promotion"[Title/Abstract] AND (2007:2023[pdat])) AND (medication[MeSH Terms])) OR ("medication"[Title/Abstract]) AND ((adult[Filter] OR middleaged[Filter]) AND (2007:2023[pdat]))) AND ("prevention"[Title/Abstract])) OR (prevention[MeSH Terms])) AND (cardiovascular disease[MeSH Terms])) OR ("cardiovascular disease"[Title/Abstract])

**Embase**

1. First search to screen primary results: 'health promotion'/exp OR 'health behavior'/exp OR 'health promotion':ab,ti OR 'health behavior':ab,ti OR 'healthy people programs':ab,ti AND 'primary health care'/exp OR 'primary medical care'/exp OR 'primary health care':ab,ti OR 'primary medical care':ab,ti OR 'primary care':ab,ti

2. (('primary health care'/exp OR 'primary medical care'/exp OR 'occupational health'/exp OR 'prevention'/exp) AND 'intervention'/exp OR 'program'/exp OR 'check') AND (('primary health care':ab,ti OR 'primary medical care':ab,ti OR 'primary care':ab,ti) AND 'occupational health':ab,ti AND 'intervention':ab,ti OR 'program':ab,ti OR 'check':ab,ti) AND [2007-2023]/py AND ([adult]/lim OR [middle aged]/lim OR [young adult]/lim)

3. (('primary health care'/exp OR 'primary medical care'/exp OR 'dental'/exp OR 'prevention'/exp) AND 'intervention'/exp OR 'program'/exp OR 'check') AND (('primary health care':ab,ti OR 'primary medical care':ab,ti OR 'primary care':ab,ti) AND 'dental':ab,ti AND 'intervention':ab,ti OR 'program':ab,ti) AND 'check':ab,ti AND [2007-2023]/py AND ([adult]/lim OR [middle aged]/lim OR [young adult]/lim)
