# Supplementary material for: Unleashing the potential of Health Promotion in primary care—a scoping literature review
Source: Health Promot Int. 2024 May 25;39(3):daae044. doi: 10.1093/heapro/daae044 (PMC11127486; doi:10.1093/heapro/daae044)
Supplement: daae044_suppl_Supplementary_Table_S1 [file daae044_suppl_supplementary_table_s1.docx]

| **Table 1: An overview and summary of the 42 studies included in the review with relevant results** | | | | | | | | |  |
| --- | --- | --- | --- | --- | --- | --- | --- | --- | --- |
| **Reference / Country** | Participants | Study design | Setting | Main target | Healthy individual participation | Type of intervention | Outcome measures | Main outcomes |  |
| **Anokye, Lord and Fox-Rushby, 2014 / UK** | A cohort of 100 000 physically inactive but healthy adults, starting age 33 | Economic evaluation | Community-based / Primary care | Lifestyle | Participants | Initial measure + brief advice, follow up of health outcomes | Health outcomes / QALYs | Brief advice in primary care is a cost-effective way to promote physical activity. It is more effective (leading to 466 QALYs gained) compared to standard care | |
| **Baumann et al., 2015 / Denmark** | An age- and sex-stratified random sample of 13,016 people, aged 30-60 | Randomised controlled trial | Community based / Primary care | Lifestyle | Participants and controls | A tailored individual (based on lifestyle and CV risk score) lifestyle counseling focusing on smoking, physical activity, diet, and/or alcohol consumption; regardless of classified as high or low risk. + group-based counseling on smoking cessation or on diet and physical activity for high-risk (intervention) group | Behavioural outcomes: Physical activity, dietary quality score Baseline health covariates: self-perceived health, smoking, alcohol consumption, physical activity during leisure time, being limited in stair climbing  self-perceived risk of IHD | Five years after the intervention, women in the intervention group reported greater improvements in the intake of fruit (MΔ = 90.2 g/week, p = 0.041) and less intake of saturated fat (OR = 0.30, 95% CI: 0.17–0.54) than the control group. Men in the intervention group reported greater improvements in physical activity (MΔ = 19.6 min/week, p = 0.003) and less intake of saturated fat (OR = 0.31, 95% CI: 0.17–0.56) than the control group | |
| **Biffi et al., 2018 / Italy** | 175 physically active workers | Case control study | Occupational /Worksite | Lifestyle | Participants | Individual supervised exercise program (2–3 times/week gym frequency) in a gym center, accessible 12 h/day, dietary counseling included | Demographic and Clinical Data (BMI, hdl, fasting glucose), Exercise stress testing data, adherence to the program | A relevant improvement of several CV risk factors (body mass index, total and LDL cholesterol, triglycerides, blood pressure) and cardio-respiratory fitness parameters (estimated VO2max, Watt peak, METs) compared to baseline values. A clear “drag effect” towards nonparticipating and sedentary peers, resulting in a 90% adherence increase over the years. | |
| **Blomstedt et al., 2015 / Sweden** | N=101918 people from Västerbotten county, age: 40, 50 or 60,  control group: reference population: Swedish population aged 40, 50 or 60 years residing in Sweden, including Västerbotten | Cohort study | Community-based | Lifestyle | Participants | Health dialogue (physical examination + comprehensive questionnaire at 40, 50, 60) A systematic long-term, county-wide cardiovascular disease (CVD) intervention, individual and population approach. | All-cause and CVD mortality | Significant impact on all-cause and CVD mortality. Compared to Sweden at large, the standardised all-cause mortality ratio was 90.6% (95% CI 88.2% to 93.0%). For CVD, the ratio was 95.0% (95% CI 90.7% to 99.4%). For participants, the standardised all-cause mortality ratio was 66.3% (95% CI 63.7% to 69.0%), whereas the CVD ratio was 68.9% (95% CI 64.2% to 73.9%). | |
| **Bo et al., 2016 / Italy** | e 45–64 years old Caucasian patients (n = 1658) | Cohort study | Community based / Primary care | Lifestyle | Participants | Health screening (medical examination), self-report questionnaire about food, blood sample analysis, follow-up - medical examination by a GP. | MED score, Framingham risk score for baseline, PA level | A high adherence to the Mediterranean nutritional pattern favourably affected life expectancy and the risk of CV events and death among middle-aged individuals with a low CV risk. In a Cox-regression model, the hazard ratios (HRs) in low-risk individuals per unit of MED score were HR = 0.83 (95 % CI 0.72–0.96) for all-cause mortality, HR = 0.75 (95 % CI 0.58–0.96) for CV mortality, and HR = 0.79 (95 % CI 0.65–0.97) for CV events, after multiple adjustments | |
| **Bonaccio et al., 2019 / Italy** | population based cohort, n = 22 839, general population from Molise region | Cohort study | Community-based | Lifestyle | Participants | Health check up, adherence to health behaviors, follow up, health-check up | Adherence to Mediterranean diet,  Smoking, Leisure-time, physical activity, A healthy lifestyle score | In the general population, adherence to all four healthy lifestyles, compared with none or 1, was associated with lower risk of all-cause (HR = 0.53; 95%CI:0.39–0.72), CVD (HR = 0.54; 0.32–0.91), cancer (HR = 0.62; 0.39–1.00) and mortality from other causes (HR = 0.39; 0.19– 0.81) Adherence to four healthy lifestyle factors substantially reduced the risk of all-cause and cause-specific mortality, both in a general adult population and among high-risk groups. | |
| **Buckland et al., 2009 / Spain** | n = 41,438 healthy volunteers, aged 29–69 years | Prospective cohort study | Community-based / Primary care | Lifestyle and diagnosis | Participants | Dietary history (last 12 months) questionnaire, lifestyle questionnaire - sociodemographic, lifestyle factors including history of tobacco use as  well as work and leisure-time physical activity, medical  history, and reproductive indicators (women), health indicators, follow up in 3 years | Adherence to a Mediterranean diet - rMED score, self-report | High compared with low relative Mediterranean diet score was associated with a significant reduction in CHD risk (hazard ratio = 0.60, 95% confidence interval: 0.47, 0.77). A 1-unit increase in relative Mediterranean diet score was associated with a 6% reduced risk of CHD (95% confidence interval: 0.91, 0.97). | |
| **Byrne et al., 2016 / Netherlands** |  | Cohort study | Community / Workplace | Lifestyle | Participants | Physical activity intervention, self-report on health behaviors, health outcome comparison with sedentary population (control) | daily modifiable behaviours, biologically plausible contributors to health outcomes, positive health behaviours (self report) | Compared with sedentary participants, those who exercised 4 days per week were less likely to develop new-onset diabetes (HR=0.31, 95% CI=0.20, 0.48); heart disease (HR=0.46, 95% CI=0.27, 0.80); and hypercholesterolemia (HR=0.61, 95% CI=0.50, 0.74). | |
| **Currat, Lazor-Blanchet and Zanetti, 2020 / Italy** | ≥18, 79 employees: 183 in the control group and 196 in the intervention group | Randomised controlled trial | Occupational /Worksite | Lifestyle | Participants | Pre-employment health check + The intervention group underwent an additional three-phase intervention - a shot, briefing, current recommendations, annual campaign, semi-structured interview about previous vaccination, answered questions from nurse | Vaccination rate | A significantly higher rate of vaccination was noted among physicians (70/117, 60%) than among other employees (101/240, 42%, p = 0.001). In a pre-defined exploratory analysis among physicians, the vaccination rate was higher in the intervention group (36/51, 71%) than in the control group (34/65, 52%, p = 0.046). | |
| **Dalager et al., 2016 / Denmark** | N = 387, training (TG) and control group (CG) | Randomised controlled trial | Occupational /Worksite | Lifestyle | Participants | The training program (TG) 1h/week for 2 years, the year 1 fully supervised, year 2 - monthly supervision of a weekly training session. Each participant in TG - tailored training program based on outcome measures of the baseline health check | Maximal oxygen uptake, blood pressure, blood profile, body composition | Adherence of ≥70 % demonstrated a significant increase in CRF of more than 10 % compared with CG, and a significant reduction in systolic blood pressure (−5.3 ± 13.7 mm Hg) compared with CG. Effectiveness of health promotion by implementing physical exercise training at the workplace | |
| **Doumas and Hannah, 2008 / USA** | 18- to 24-year-old, N = 196, mostly female | Randomised controlled trial | Occupational /Worksite + Web based | Lifestyle | Participants | Participants randomly assigned into three groups: web-based personalized feedback program alone (WI), web-based personalized feedback program with a 15-minute motivational interviewing session (MI), or the control group. | Several measures of alcohol use, descriptive drinking norms | Reductions in the intervention group were significantly greater than those in the control group for weekend drinking, frequency of drinking to intoxication, and peak consumption. Intervention group high-risk drinkers had greatest decreases in drinking between baseline and the 30-day follow-up assessment. Program was most effective in reducing drinking for young adults who reported high-risk drinking at the baseline assessment | |
| **Eng, Moy and Bulgiba, 2016 / Malaysia** | Age >= 35, N = 1365 | Cohort study | Occupational /Worksite | Lifestyle / diagnosis | Participants | Low intensity program, annual health screening and physical examination, health educational seminars and health exhibitions. -Healthy diet, physical activity, smoking and stress management. Employees who found to be at risk of obesity, hypertension, hypercholesterolemia and diabetes followed up with face-to-face lifestyle counselling conducted by dietitians, or referral for medical treatment when necessary. | A self-administered questionnaire, weight, BMI, smoking, resting blood pressure | Significant decrease in systolic (-0.75 mmHg, p<0.001) and diastolic (-0.56 mmHg, p<0.001) blood pressure in hypertension risk group, no significant change among participants in the healthy subgroup over the 6-year period. | |
| **Gibson et al., 2014 / Ireland** | N= 521 patients, mean age 57.5 | Observational study | Community-based | Lifestyle / diagnosis | Participants and controls | "Individual approach: -Motivational interviewing and stages of change assessment technique, Goal Setting, Personal Record Card -Weekly exercise class and educational workshops on CVD, Risk factors for CHD and Stroke, -Healthy Eating and Alcohol, Healthy Eating and Weight Management, Physical Activity, Stress Management, Food Labels, Maintaining Change, Cardiac Medications  -Serial BP, BMI, lipid and glycaemia measurements with continued goal setting  -Weekly meetings  -Targeted and protocol driven pharmacotherapy to supplement lifestyle changes." | Smoking habit, diet, food habit questionnaire, Mediterranean diet score, physical activity levels, functional capacity, weight, BMI, waist circumference, psychosocial measures, blood pressure, fasting lipids and glucose, use of cardio medications | There were statistically significant improvements in both lifestyle (body mass index, waist circumference, physical activity, mediterranean diet score, fish, fruit, and vegetable consumption, smoking cessation rates), psychosocial (anxiety and depression scales and quality of life indices), and medical risk factors (blood pressure, lipid and glycaemic targets) between baseline and end of programme, with these improvements being sustained at 1-year follow up. | |
| **Gram et al., 2012 / Denmark** | Exercise (N = 35), control (N = 32) | Randomised controlled trial | Occupational / Worksite | Lifestyle | Participants and controls | "Individual exercise intervention (during working hours, the training implemented in cooperation with the employer. 1h/week (3 × 20 minutes) for 12 weeks, supervised by skilled instructors in two of the three weekly sessions, Each participant received their own individual exercise protocol in a training diary that had to be completed at each training session  - health check before and after (measures of VO2max, isometric muscle strength, body mass, percent fat, blood pressure, and blood lipid profile)" | Primary outcome variables: VO2max and isometric muscle strength. Secondary outcomes: BMI, fat percent, blood pressure, and blood lipid profile. | Group x time analyses showed a significant difference in estimated change in VO(2max) of 0.4 l/min for the exercise group and 0.0 l/min for the control group (P<0.001). Training for 20 minutes, 3 times a week significantly increased VO(2max) with a clinically relevant magnitude regarding risk of cardiometabolic disorders. | |
| **Grunfeld et al., 2013 / Canada** | N = 789, age 40 - 65 | Randomised controlled trial | Community-based / Primary care | Diagnosis | Participants and controls | "Practice-level intervention with a Practice Facilitator and a patient-level intervention with a Prevention Practitioner:  1) Participation in the Clinical Working Group 2) a two-day training workshop, followed by a one-day training workshop before the intervention; and 3) during the intervention - the opportunity to participate in one-hour monthly teleconferences that were facilitated by a member of the Clinical Working Group. " | EMR, health survey, summary quality index | Prevention Practitioner, taken from within the practice and trained to conduct dedicated prevention visits at which each patient was given a tailored prevention prescription and directed to relevant practice or community resources, improved Chronic disease prevention and screening by 32.5% compared to control. Benefits seen both in general and mental health strata. | |
| **Hardcastle, Blake and Hagger, 2012 / UK** | N = 207, clinic patients | Non-randomised experimental study | Community-based / Primary care | Lifestyle | Participants | Individual approach: lifestyle change facilitation service - motivational interviewing + transtheoretical model, reason for change not discovered to patients (building autonomous motivation) | PA (IPAQ), Psychological variables (Stages of change flow chart, Self efficacy, Behavioural regulation in exercise, attitude, perceived behavioural control, social support, health care climate questionnaire), SES | There were significant increases in total physical activity, walking, vigorous physical activity, stage of change, and social support from baseline to follow-up | |
| **Hjarnoe and Leppin, 2013 / Denmark** | N = 343 who filled the questionnaire, age mean 42 years | Non-randomised experimental study | Occupational / Worksite | Lifestyle | Participants | 5 interventions: cooking course (all trained and untrained ship cooks), upgrade of fitness room facilities (all ships in need), group based smoking cessation, individual exercise guidance, individual health check-up The questionnaire in 2007 and one year after, to the home address of all seafaring employees, electronic version for a follow-up | Standardized questionnaire, individual health profile: anthropometric and cv fitness measurement | Significant changes were identified for levels of fitness, daily sugar intake and metabolic syndrome. However, these results were not associated with participating in the health educational interventions | |
| **Jeong et al., 2019 / Korea** | N = 441 798, population-based cohort. | Cohort study | Community-based / Primary care | Lifestyle and diagnosis | Participants | Physical activity (self-report) and health check the only intervention, follow-up | All-cause mortality, cardiovascular death and non-cardiovascular death | The benefit in the secondary prevention group (with CVD) was shown to be greater than that in the primary prevention group: every 500 MET-min/week increase in physical activity resulted in risk reduction in mortality in the secondary (14%) and primary prevention (7%) groups, (interaction P < 0.001). Individuals with CVD may benefit from physical activity to a greater extent than do healthy subjects without CVD. | |
| **Journath et al., 2020 / Sweden** | (n=5761) Sollentuna inhabitans who visited a primary healthcare centre and agreed to participate in the SoPP | Observational study | Community based / Primary care | Lifestyle and diagnosis | Participants and controls | Individualised and customised advice, large selection of different educational groups and a lecture series were available as support, introduction of physical activity on prescription program (health promotion of a physically active lifestyle), measures of CV and mortality risk | A CV event, CV death, all-cause death | The HR (95% CI) in the intervention group compared with the reference group was 0.88 (0.81 to 0.95) for first CV events, 0.79 (0.70 to 0.89) for CV deaths and 0.83 (0.78 to 0.89) for all-cause deaths. Participation in a CVD prevention programme in primary healthcare focusing on promotion of physical activity and healthy lifestyle was associated with lower risk of CV events (12%), CV deaths (21%) and all-cause deaths (17%) after two decades | |
| **Khadjesari et al.,2014 / UK** | n = 3375, experimental group, control group, and feedback only on health behaviors group | Randomised controlled trial | Occupational / Worksite | Lifestyle | Participants and controls | The core intervention: screening and personalised feedback, the option of a more extended intervention for those who wanted.  Intervention: feedback on all health behaviours assessed in the health check, alcohol feedback if they reached a score 5, if not - excluded and got feedback on other health behaviors  Brief advice on alcohol behavior: Feedback, information and a hyperlink to website Down Your Drink (DYD), -a resource for participants who wanted help to reduce their drinking  Control group: feedback on all health behaviors except alcohol consumption. At the follow-up after three months everybody in the experimental arm - feedback on alcohol consumption. | Primary: TOT-AL, an online measure of self-reported past week alcohol intake Secondary: AUDIT (past three months frame), Health state (EQ-5D), number of days of sickness absence in the past three months (Self-report), primary and secondary health care resource use (self-report) | At three-month follow-up, there was no statistically significant difference in past week alcohol consumption between employees who did and did not receive online personalised feedback on their drinking in the context of a health check. | |
| **Kirkman, Leo and Moore, 2018 / Australia** | 1917 adults, average 46, Australia, US, UK, Canada | Observational study | Community / Web based | Lifestyle | Participants | Blogging forum web-based intervention,  -Hello Sunday Morning (HSM) - social media health promotion “movement” that asks participants to publicly set a personal goal to stop drinking or reduce their consumption, for a set period, and to record their reflections and progress on blogs and social networks  -Blogging, social media, and gamification | Change in Alcohol Use Disorders Identification Test (AUDIT) scores | Individuals who reported hazardous (pre-program AUDIT mean 11.92, SD 2.25) and harmful consumption levels (pre-program AUDIT mean 17.52, SD 1.08) and who engaged in the HSM program reported a significant decrease in alcohol consumption, moving to lower risk consumption levels (hazardous, mean 7.59, SD 5.70 and harmful, mean 10.38, SD 7.43), 4 months following program commencement (P<.001). Those who reported high-risk or dependent consumption levels experienced the biggest reduction. | |
| **Kosendiak, Felińczak and Szymańska-Chabowska, 2021 / Poland** | 76, men and women, participants of you can be a marathon runner too programme | Observational study | Community-based | Lifestyle | Participants | Screening, marathon preparation, screening, biochemical and health measures of interest | Blood test results | The BMI decreased from 25 kg/m2 to 23 kg/m2 and the per- centage of body fat - from 25% to 21%. Furthermore, some blood parameters decreased: cholesterol from 217mg/dL to 196mg/dL, triglycerides from 128 mg/dL to 97 mg/dL, and glucose from 82 mg/dL to 79 mg/dL. Physical activity can decrease CVD risk | |
| **Lidin et al., 2018 / Sweden** | n=100 men and women ≥ 18 year presenting at least three risk factors | Prospective observational intervention | Community-based / Primary care | Lifestyle and diagnosis | Participants | Individual + group-based approach -An individual visit to a nurse at baseline, at 6 months and 1 year. The  nurse preformed a health check-up with a person-centred approach using motivational technique for support in behavioural change of unhealthy lifestyle habits. A personalized prescription of PA and a pedometer given. After the initial visit, participants were offered participation in five educational group sessions led by a nurse and a physician with focus on lifestyle habits; 1) overall lifestyle and health 2) physical activity and sedentary behaviour 3) dietary habits and use of alcohol and tobacco 4) stress and sleeping habits and 5) behavioural change.  + free web-based lifestyle course | CVD risk factors | Cardiovascular risk decreased significantly from baseline (15.6%) to the one-year follow-up (13.3%) in the total study population, corresponding to a 15% decrease over 1 year. Significantly lower waist circumference, systolic BP, diastolic BP and total cholesterol over 1 year in the participants enrolled in the lifestyle program. Probability of developing a CVD within 10 years decreased by 15% according to the cardiovascular risk based on Framingham risk-score. | |
| **Lingfors et al., 2009 / Sweden** | Inhabitants in four communities in Skaraborg at ages both 30 and 35 - target group, other four communities only at age 35 - reference group | Cohort study | Community-based | Lifestyle | Participants and controls | Individualised health intervention programme and advice for physical activity (and health), diet, mental health and hereditary diseases, health-check measures and a follow-up | Health and risk factors for IHD in the Health Curve including tobacco, alcohol, diet, physical activity, psychosocial strain, mental stress, body mass index (BMI), waist-hip-ratio (WHR), serum cholesterol, blood pressure, chronic disease, and heredity for diabetes and cardiovascular disease | Reference group at the age of 35 showed significantly worse health outcomes compared to target community. The proportion of persons with BMI ≥ 25 increased with 26.4% (absolute change 9.6%) and the proportion with elevated waist circumference increased with 39.3% (absolute change 11.6%). The proportion of persons with cholesterol ≥ 5 mmol/L increased with 20% (absolute change 10.4%)  Inhabitants in communities where there had been a previous individualised health intervention programme had, on the community level, a more favourable development concerning dietary habits, mental stress, BMI, waist circumference, cholesterol, blood pressure and metabolic risk profile compared to inhabitants in communities with only a community-based health intervention programme. Looking at a metabolic risk profile, proportion had increased with 17.4% (absolute change 2.8%) in the reference communities and had decreased with 42.3% (absolute change 3.3%) in the target communities between the two study periods, with the change being highly significant. | |
| **Lingfors and Persson, 2019 / Sweden** | 652 men 33-42, living in Habo community | Longitudinal follow-up register study | Community-based | Lifestyle and diagnosis | Participants | Building an individual health-profile, regular check-ups, self report measures on health habits + health measures and a follow-up | Health profile and self report health habits, biological risk markers | At follow-up, all-cause mortality was 29% lower (OR=0.71,95% CI 0.53 to 0.95) among all men invited to the health dialogue compared with all men from the same age cohort in all of Sweden (intention-to-treat) and 43% lower (OR=0.57, 95% CI 0.40 to 0.81) among participating men (on-treatment). A healthy lifestyle was associated with lower mortality (OR=0.16, 95% CI 0.07 to 0.36), with the strongest association for no smoking (OR=0.38, 95% CI 0.21 to 0.68) and a healthy diet (OR=0.37, 95% CI 0.20 to 0.68). A healthy lifestyle was also associated with a decreased incidence of CVD and cancer | |
| **Matano et al., 2007 / USA** | 229 employees that logged on the website | Pilot study | Worksite / Web based | Lifestyle | Participants and controls | Web-based; Assessed employee’s alcohol use pattern, to help employees reduce their quantity and frequency of alcohol consumption, and to prevent both moderate- and low-risk drinkers from developing alcohol-related problems. | Demographics, assessment of alcohol-related problems, alcohol consumption, experiences in using website | Greater alcohol reduction among participants who received full individualized feedback, although sample size was inadequate for evaluating treatment effects on drinking. | |
| **Matzer et al., 2018 / Austria** | n = 89, 81 after dropping out, The mean age was 44 years | Randomized cross-over trial | Occupational / Worksite | Lifestyle | Participants | The four stress‐relieving interventions included two interventions that combined physical activity and relaxation (1 and 2) and two interventions that consisted of relaxation only (3 and 4): (1) moderate physical activity (walking) combined with resting, (2) moderate physical activity (walking) combined with balneotherapy, (3) combined relaxation (resting and balneotherapy), and (4) resting only | Salivary cortisol as a primary outcome measure; secondary outcome measures were blood pressure, mood, and subjective level of relaxation | The systolic blood pressure was reduced best when walking was combined with balneotherapy or resting (F = 7.34; p < .001). Participants with high stress levels (n = 25) felt more alert after interventions including balneotherapy, whereas they reported an increase of tiredness when walking was combined with resting (F = 3.20; p = .044). Results suggest that combining physical activity and relaxation (resting or balneotherapy) is an advantageous short-term strategy for stress reduction | |
| **McEachan et al., 2011 / UK** | N = 1260, measured for at least one time point | Randomized controlled trial | Occupational / Worksite | Lifestyle | Participants and controls | The form of an easy to implement toolkit, delivered in-house by trained local facilitators, physical activity intervention, measures of health and fitness | Primary outcome measure: Moderate - Vigorous MET minutes of Physical Activity, Secondary outcome measures: Objective measures of health and fitness and other questionnaires | The intervention significantly reduced systolic blood pressure (B=-1.79 mm/Hg) and resting heart rate (B=-2.08 beats) and significantly increased body mass index (B=.18 units) compared to control. | |
| **Neuner-Jehle, Schmid and Grüninger, 2013 / Switzerland** | n = 1045 patients, recruited by 20 GPs. The mean age of patients was 50 (range 15–75) years. Representative for average population of Switzerland | Observational study | Community based | Lifestyle | Participants | “Health Coaching” project  -A change of roles and sharing responsibility: Patient and GP are a team  -Patient-centred choice of the area of action: dietary habits and body weight control, physical activity, smoking, alcohol consumption, and psychosocial stress  -The counselling techniques: health literacy and patient empowerment, shared decision making, transtheoretical model of behaviour change (TTM), counselling based on motivational stages, motivational interviewing, and various risk communication formats and models  -GP training courses | Participation rates; the duration of counselling; patients’ self-rated behavioural change in their areas of choice; and ratings of motivational, conceptual, acceptance, and feasibility issues | The proportion of favourable health behaviour ratings increased from 9% to 39%. The ratings for motivation, concept, acceptance, and feasibility of the “Health Coaching” programme were consistently high. The program is highly feasible in primary care | |
| **Nguyen et al., 2012 / Vietnam** | n = 4,650 adults above 25 years old | Quasi-experimental | Community based / Primary care | Lifestyle and diagnosis | Participants | Two integrated components simultaneously targeting two different groups: (1) hypertensives only, with monthly check-ups to control their blood pressure with multidrug therapy + lifestyle modifications and individual advice; (2) healthy adults - lifestyle promotion campaigns via broadcasting, leaflets or meetings | Changes in CVDRF patterns, especially mBP changes | A significant reduction in systolic and diastolic BP (3.3 and 4.7 mmHg in women versus 3.0 and 4.6 mmHg in men respectively) in the general population at the intervention commune. Health promotion reduced levels of salty diets but had insignificant impact on the prevalence of daily smoking or heavy alcohol consumption | |
| **Oude Hengel et al., 2014 / Netherlands** | intervention group, n = 171 and control group n = 122, construction workers | Economic evaluation of a randomized controlled trial | Occupational / Worksite | Lifestyle | Participants and controls | Two individual training sessions with a physical therapist to reduce physical pain, an instrument to raise awareness of the importance of rest breaks to reduce fatigue (Rest-Break tool), and two empowerment training sessions to improve the range of influence at the worksite | Primary outcomes: work ability, physical and mental health, the secondary outcomes included the prevalence of musculoskeletal symptoms in different body regions | After 12 months, the absenteeism costs were significantly lower in the intervention group than in the control group, intervention was found cost-saving to the employer | |
| **Pemberton et al., 2011 / USA** | Control group (n = 914) or to one of the program conditions (AS [n = 686] or DCU [n = 1,470]), military personnel | Evaluation of two interventions | Worksite / Web based | Lifestyle | Participants and controls | Two web-based interventions and the comparison:  -Alcohol Savvy (AS) - alcohol-misuse-prevention program initially developed for adults in the workplace  -Drinker’s Check-Up (DCU) - a brief motivational intervention | Measured at baseline, one, and six months: Average number of days that alcohol was used, Average number of drinks consumed per drinking occasion, Number of days perceived drunk, Heavy episodic drinker status, Heavy episodic drinking episodes, Frequent heavy episodic drinker status, estimated peak BAC, Control measures | At 1-month follow-up, participants who completed the Drinker's Check-Up intervention had significant reductions in multiple measures of alcohol use relative to controls, no significant changes found with Alcohol Savvy | |
| **Persson et al., 2015 / Sweden** | n = 757 men 33-42, living in Habo community | Follow-up study of a cohort | Community based / Primary care | Lifestyle and diagnosis | Participants | Screening/ health profile and dialogue, individual doctor advice for high risk | CVD and cancer diagnoses | Risk factors measured on one occasion seemed to be able to predict CVD, cancer and diabetes 26 years later. | |
| **Recio-Rodriguez et al., 2016 / Spain** | n = 833 recruited in six primary care centres | Randomized controlled trial | Community-based / Primary care + Mobile App | Lifestyle | Participants but with different interventions | Counselling on PA and the Mediterranean diet- both groups  -The App + counselling participants - training in the use of an app designed to promote PA and the Mediterranean diet over a 3-month period.  -A research nurse- intervention lasting 30 minutes in both groups: standardized counselling in PA and the Mediterranean diet, leaflets. | The main outcome measures: change in PA and adherence to the Mediterranean diet at 3 months in the app+counselling group compared to the counselling only group. Other outcome measures: blood pressure, waist circumference, body mass index (BMI), and laboratory parameters | Leisure-time moderate-to-vigorous physical activity (MVPA) by 7-day PAR increased in the app+counselling (mean 29, 95% CI 5-53 min/week; P=.02) but not in the counselling only group (mean 17.4, 95% CI –18 to 53 min/week; P=.38). Adherence to the Mediterranean diet increased in both groups (8.4% in app+counselling and 10.4% in counselling only group), with an increase in score of 0.42 and 0.53 points, respectively (P<.001), but no difference between groups (P=.86). | |
| **Richardson et al., 2008 / UK** | n = 596, men and women aged between 45 and 64 | Evaluation of an intervention | Community-based / Primary care | Lifestyle and diagnosis | Participants | A community-based intervention, advice + screening (primary prevention of CHD), follow up | The mean reduction in the Framingham risk score | The mean reduction in the Framingham risk score, was significantly lower at one year (0.876, 95% CI 0.211 to 1.541, p = 0.01). The mean 10-year risk of CHD at baseline was 13.14% (SD 9.18) and had fallen at follow-up to 12.34% (SD 8.71), a mean reduction of 6.7% of the initial 10-year Framingham risk. | |
| **Robroek et al., 2010 / Netherlands** | n = 726 employees | Observational study of several RCTs | Worksite / Web based | Lifestyle | Participants | Individualized: Website: baseline questionnaire, appointment for a physical health check. The health check at the workplace. Immediately after all participants received results in print. These results were discussed, individual advice on how to improve or maintain their lifestyle individually.  -Participants who were prehypertensive or who had an elevated cholesterol level were advised to visit their general practitioner or the occupational physician  -After health checks, all participants were invited visit the website to view their health check results and the personal advice | Demographic characteristics, lifestyle behaviour and health indicators, physical health check, social cognitive variables | individuals with an elevated total cholesterol level visited the website more often (OR 1.44, 95% CI 1.05-1.98). Participants with a positive attitude toward increasing physical activity were less likely to visit the website (OR 0.54, 95% CI 0.31-0.93) or to use the self-monitor and FFQ (OR 0.50, 95% CI 0.25-0.99). Healthy individuals were not found more susceptible to HP. | |
| **Van Doorn et al., 2019 / Ireland** | n = 310, convenience sample of farmers | Evaluation of an intervention | Occupational / Worksite | Lifestyle and diagnosis | Participants but 2/3 referred to GP, others got only a brief intervention | Screening, individual brief health intervention, screening | Biological and self-report | At Week 12, 48% reported having changed at least one health behaviour. The majority of farmers were referred to their GP and by Week 12, 32% had acted on this advice. Participants most in need of health behaviour change based on adverse health screening results were, paradoxically, the least likely to contemplate or engage in health behaviour change. | |
| **Viester et al., 2015 / Germany** | intervention group (n = 162) receiving personal coaching, tailored information and materials, and a control group (n = 152) receiving usual care, randomized, construction workers | Randomized controlled trial | Occupational / Worksite | Lifestyle | Participants and controls | Individual lifestyle coaching programme tailored to the participant’s weight status, physical activity level, and stage of change. The intervention programme focused on improving (vigorous) physical activity levels and healthy dietary behaviour. Tailored lifestyle information, lifestyle coaching sessions, exercise instructions, and the ‘VIP in construction toolbox’. + personal energy plan | musculoskeletal symptoms, physical functioning, work-related measures | The intervention improved physical activity, dietary, and weight-related outcomes, | |
| **Watson et al., 2015 / UK** | 627 employees screened, 163 (26.01%) fulfilled the inclusion criteria, 57 accepted | Randomized controlled trial | Occupational / Worksite | Lifestyle | Participants only hazardous drinkers, others excluded | Individual; The participants randomised to the experimental group and received the brief intervention: a one-to-one consultation during which information aimed at promoting behaviour change was provided and was underpinned by self-efficacy and readiness to change theory | primary: AUDIT score, secondary: changes in the quality of life and economic indicators in terms of service use, employment outcomes, public sector resource and employment costs as measured by the EQ-5D | A statistically significant effect was found in the mean AUDIT scores over time (F=8.96, p=0.004). Potential of brief workplace interventions to reduce alcohol related harm and save public sector resources. | |
| **Wendel-Vos et al., 2009 / Netherlands** | Aged 20–59 years inhabitants of Maastricht or Doetinchem The study population consisted of 3114 men and women (intervention: n = 2356; control area= 758) aged 31–70 years | Cohort study | Community-based | Lifestyle and diagnosis | Participants and controls | Major interventions: nutrition parties; debt assistance (people with debts are taught to cook a healthy meal on a small budget); printed guides showing walking and cycling routes; a daily TV guided-aerobics program, including information about the health advantages of exercising;  Two strategies: a population strategy aimed at all inhabitants and, specifically, at groups with low SES, and a high-risk strategy that focused on individuals diagnosed with CVD or with multiple biological risk factors for CVD | Lifestyle factors | The adjusted difference in mean change in lifestyle factors between the intervention group and the control group was significant (p<or=0.05) for energy intake, fat intake, time spent walking, time spent on total leisure-time physical activity, and time spent bicycling. Lifestyle factors changed unfavourably in the control group, whereas changes were less pronounced or absent in the intervention group. | |
| **Yang and Kim, 2022 / Korea** | Korean women aged 40–59 years in the community experimental group (n = 22), control group (n = 22) | Non-randomised experimental study | Community-based | Lifestyle | Participants and controls | Pretest, health check, self-report questionnaire, walking excercise focused health promotion program | Pretest, health check, self-report questionnaire, walking exercise focused hp programme | After the intervention, in the experimental group, the level of physical activity (Z = −2.065, p = 0.039) and health-promoting lifestyle improved (t = 3.344, p = 0.002), and both waist circumference (t = −4.328, p < 0.001) and perceived stress (t =−3.578, p < 0.001) decreased. | |
| **Zabaleta-Del-Olmo et al., 2021 / Spain** | n = 1481 randomised to the intervention and n = 1581 participant control group (usual care), age: 45-75 | Randomized controlled trial | Community-based / Primary care | Lifestyle | Participants and controls (usual care) | Transtheoretical Model (TTM) and Stages of Change by PHC professionals, screening + assessing target behaviors and stages of change.  -Behavioral screening: smoking, fruit and vegetables, brief physical activity assessment tool  -Individual approach: brief intervention to raise awareness, prevention, plan  -The group approach: health education workshops on healthy diet and physical activity,healthcare professionals  -The community approach - mainly social prescription of resources and activities offered in the participants’ communities  -Control group: Preventative activities and health promotion, systematic screening, brief advice on CVD, mental disease, cancer and vaccines | Positive change in physical activity behaviour: sufficient physical activity in previously insufficiently active people. The International Physical Activity Questionnaire, Positive change in dietary behaviour: adherence to a Mediterranean dietary pattern in people with low adherence at baseline. The 14-item Questionnaire of Mediterranean Diet Adherence | 14.5% of participants in the intervention group and 8.9% in the usual care group showed a positive change in two or all the target behaviours. Intervention was more effective in promoting dietary behaviour change (31.9% vs 21.4%). | |
